# Capacity-Building for Collecting Patient-Reported Outcomes and Experiences (PRO) Data Across Hospitals

**DOI:** 10.1007/s10995-023-03720-6

**Published:** 2023-06-22

**Authors:** Samia Saeb, Lisa M. Korst, Moshe Fridman, Jeanette McCulloch, Naomi Greene, Kimberly D. Gregory

**Affiliations:** 1 Department of Obstetrics and Gynecology, Cedars-Sinai Medical Center, Los Angeles, CA USA; 2 Maternal Metrics, Inc, North Hollywood, CA USA; 3 BirthSwell, Ithaca, NY USA

**Keywords:** Patient-reported outcomes, Childbirth experiences, Implementation science, Clinical informatics, PRO data collection, Case study, Learning collaborative, Patient satisfaction

## Abstract

**Purpose:**

Patient-reported outcomes and experiences (PRO) data are an integral component of health care quality measurement and PROs are now being collected by many healthcare systems. However, hospital organizational capacity-building for the collection and sharing of PROs is a complex process. We sought to identify the factors that facilitated capacity-building for PRO data collection in a nascent quality improvement learning collaborative of 16 hospitals that has the goal of improving the childbirth experience.

**Description:**

We used standard qualitative case study methodologies based on a conceptual framework that hypothesizes that adequate organizational incentives and capacities allow successful achievement of project milestones in a collaborative setting. The 4 project milestones considered in this study were: (1) Agreements; (2) System Design; (3) System Development and Operations; and (4) Implementation. To evaluate the success of reaching each milestone, critical incidents were logged and tracked to determine the capacities and incentives needed to resolve them.

**Assessment:**

The pace of the implementation of PRO data collection through the 4 milestones was uneven across hospitals and largely dependent on limited hospital capacities in the following 8 dimensions: (1) Incentives; (2) Leadership; (3) Policies; (4) Operating systems; (5) Information technology; (6) Legal aspects; (7) Cross-hospital collaboration; and (8) Patient engagement. From this case study, a trajectory for capacity-building in each dimension is discussed.

**Conclusion:**

The implementation of PRO data collection in a quality improvement learning collaborative was dependent on multiple organizational capacities for the achievement of project milestones.

## Significance

Patient-Reported Outcomes (PRO) are measures of a patient’s health status. PROs are self-reported and do not require interpretation by the provider. PROs are being collected by many healthcare systems as a patient-centered approach to measuring and reporting healthcare quality. This case study outlines the critical issues involved in collecting PRO data and identifies the factors that facilitate capacity-building for PRO data collection. It provides an example of how the PRO data could be applied to improve patient satisfaction with the childbirth hospital experience.

## Introduction

Patient-reported outcomes and experiences (PRO) data are an integral component of health care quality measurement (Cella et al., 2015) and PROs are now being collected by many healthcare systems. These data have produced valuable insights at multiple levels, e.g., for patient-provider interactions and decision-making, for informing and monitoring quality improvement (QI) activities within hospitals, and for comparisons across hospitals (Black et al., 2016; Lavallee et al., 2016; Marshall et al., 2021).

The capture of PRO data for comparisons of the patient experience across hospitals has occurred through condition-specific registries that allow patient contact for this purpose (Helsten et al., 2016; O’Connor et al., 2021; Haider et al., 2020; Zheng et al., 2014); in turn, these data may be linked to clinical data and used for research or QI activities (Auffenberg et al., 2021; Marshall et al., 2021).

Despite its importance, the collection of PRO data is often challenging (Bingham et al., 2016; Lavallee et al., 2016; Nordan et al., 2018). It relies on multiple organizational capacities (i.e., resources, capabilities and competencies) and includes strong incentives and leadership, and advanced electronic processes for collecting and linking patient data from multiple sources (Korst et al., 2008). Given that the capacity to collect PRO data is fundamental to the national vision of having PROs integrated within the electronic health record (EHR) (Snyder et al., 2017), further research is needed. We sought to identify the organizational capacities for PRO data collection required by hospitals participating in a nascent QI learning collaborative that has the goal of improving the childbirth experience across multiple hospitals and hospital systems. Childbirth is a highly preference-sensitive condition (Wennberg et al., 2002), and measurement of the childbirth experience is highly reliant on PROs (Nilver et al., 2017). The focus here is on the implementation of the PRO data-collection process in which patients express their values and preferences (V&P) antepartum and report their outcomes (PROs) postpartum; the intervention being evaluated is the collection of these V&P and PRO data.

### Project Description

This project involved the implementation of the Childbirth Experience Survey (CBEX) in 16 childbirth hospitals in California from 2018 to 2021. CBEX was developed and implemented by the Childbirth Patient-Reported Outcomes Partnership and includes 18 domains relevant to the childbirth experience (Gregory et al., 2019; Korst et al., 2018). In CBEX part 1, administered in the last month of pregnancy, patients reported the aspects of the childbirth experience that they felt were important (V&P). In CBEX part 2, patients reported what happened to them (PROs) and their satisfaction with that care.

CBEX was administered online through a digital perinatal healthcare coordinator that provided a secure platform to consent and register patients for the research study and to collect and export deidentified data to the research team. Patients were offered a small incentive ($5 gift card) for each of the surveys completed.

In 2018, the research team worked with participating hospitals to assure that Institutional Review Board (IRB) requirements were fulfilled and that recruitment strategies were adapted and implemented. Enrollment was paused for most of 2020 because of the COVID-19 pandemic. In 2021, the data were exported to the research team for analysis, and a benchmarking report of deidentified results was developed.

## Methods

This study was approved by the lead IRB under Pro00050845. We used qualitative descriptive case study methodology (Yin, 2018). The authors reviewed all archival documents associated with the project, attended weekly meetings for troubleshooting and monitoring, and observed final meetings between the research team and participating hospital staff. The data sources that were used to examine the objectives and assess the progress of the project with respect to its milestones are listed in Table 1, which also provides a summary of the evidence used to evaluate the importance of organizational capacities by project milestone.

**Table 1:** Data sources as evidence for the association between organizational capacities/incentives and project milestones

Capacities	Milestones	System design	System development and project operations	Implementation
	Agreements			
Leadership	1,2,3,4,5,6,7,12,18,22	7	8	10,11,12,14,21,22
Policies/Institutional Review Board (IRB)	4,5,7	4,5,7,19	4,5,7	4,5,7
Operational systems	7	7,8,21	7,10,11	7,17,20
Information Technology (IT)	2,3,6	9,12,13,19	9,12,13	9,10,11,12,13
Legal	6			
Cross-organizational collaboration	4,5		20	7,8,14,20
Patient impact and engagement		9,13	7,9,13,17,20	17,18,14,15,20
Incentives	14,15,16,21	9,13	9,13,22	7,14,15,17,18,21,22

1. Contract with Lead Hospital and funder

2. Contract with Lead Hospital and third-party data collector #1

3. Contract with Lead Hospital and third-party data collector #2

4. Institutional Review Board (IRB) documentation from Lead Hospital

5. Institutional Review Board (IRB) documentation from participating hospitals

6. Business Associate Agreement (BAA) between third-party data collector #2 and participating hospitals

7. Weekly team meeting minutes, including Childbirth Patient-Reported Outcome (PRO) 8. Partnership monthly and quarterly meeting minutes

8. Recruitment materials for all participating hospitals

9. Childbirth Experience Survey (CBEX) hospital-based survey, pilot data, and recruitment tracking

10. Interim data analysis and report #1

11. Interim data analysis and report #2

12. Critical incidents list for hospital-based survey

13. CBEX Covid-19 survey, pilot data, and recruitment tracking

14. Presentations to potential hospitals, organizational partners, and funders

15. Presentations for academic conferences

16. Proposals to potential funders, including for collaborative funding

17. Final data analysis and reports to participating hospitals

18. Initial interviews with key informants

19. Readiness interviews

20. Interim interviews regarding recruitment approaches and other opportunities

21. Post-project interviews with key informants

22. Budget and expense reports

We used a modified conceptual framework detailed by Korst et al. to guide our data collection efforts (Korst et al., 2008). This framework hypothesizes that adequate organizational incentives and capacities allow successful achievement of project milestones in a collaborative setting. In addition to incentives, organizational capacities were classified as: Leadership, Policies/IRB, Operating systems, Information technology (IT), Legal aspects, Cross-collaboration, and Patient impact and engagement. The 4 project milestones considered here were (1) Agreements; (2) System Design; (3) System Development and Operations; and (4) Implementation.

Hospitals were initially approached based on their relationship with members of the research team. Team members visited sites and conducted readiness assessments based on the Consolidated Framework for Implementation Research (Damschroeder et al., 2009). Over a 3-year period, 24 hospitals with diverse patient populations were approached, and, of these, 16 enrolled patients.

To evaluate the success of reaching each milestone, critical incident tracking was used to determine the capacities and incentives needed to resolve them (Neale, 2008). Critical incidents were defined as problematic situations that occurred at either the project or hospital level. Tracking these critical incidents and how they impacted the success of meeting milestones provided the evidence for our evaluation of the organizational capacities needed for success.

## Results

Participating sites received a CBEX hospital report that provided information for identifying hospital best practices and developing future goals for QI initiatives. Critical incidents were organized by milestone (Table 2). These critical incidents and the data associated with them were used to document the sequence of events involved in the implementation and to explore why milestones were or were not met by each participating hospital and by the project as a whole. Several of the critical incidents are discussed below by milestone.

**Table 2:** Critical incidents, workarounds, and outcomes by milestones

Critical incidents organized by milestone	Workarounds	Outcomes
Milestone 1: agreements		
Agreements: contracts Lead hospital and funder: delay in signing contracts for administrative reasons.	Team proceeds with onboarding clinical sites pending funding availability	Contract execution and funding were delayed by 5 months.
Lead hospital and IT vendor: needed to change vendor because of requirement for personal data collection from respondent, specifically birth date.	New vendor acquired. Extensive conversations between team and vendor to communicate anticipated details of patient data collection and security requirements.	For security reasons, vendors may base their platforms on multiple pieces of personal data. A proportion of the patient population for this project were immigrants. To optimize recruitment, the Childbirth Partnership advised limiting personal data collection. Team required that IT vendor be responsible for any PHI breaches.
Agreements: hospitals Some hospitals with prior verbal agreements never joined the project, with some staff citing “survey fatigue.” There were multiple issues with hospital personnel on medical or maternity leave or changing responsibilities (getting promotions). Some hospitals required separate BAA with IT vendor.	Delays in onboarding sites were accommodated where possible. Staggered on-boarding was done intentionally to allow time for the team to work with each site. Sites without sufficient interest/follow-up, or adequate readiness were dropped. Multiple strategies were used to onboard hospitals, including: Readiness assessments On-site kick-off meetings IRB assistance BAA assistance	The project team worked primarily with physician and nurse leadership in hospital obstetrical departments and hospitals were not required to have formal agreements. There was no payment to hospitals. Because of the need for assistance from the study team, hospitals that were in the same region were better able to participate and to receive assistance than hospitals that were farther away. For example, the program manager could visit local hospitals and meet with site leads locally, but it was more difficult with outlying hospitals. This was exacerbated by the COVID-19 pandemic.
Agreements: IRB Several hospitals did not have internal IRB or the capacity to work with the lead IRB. Several hospitals had limitations placed on IRB, including no enrollment of teens, or mention of birth control. Several hospitals needed to train staff for IRB certification. Constant revisions and amendments required to IRB process and to consent forms; these needed to be incorporated at all hospitals. Some hospitals wanted to link to clinical data. Lead IRB had changing personnel. Lead IRB policies evolved over time.	The initial plan to recruit from pre-registration or postpartum database lists could not be put into place initially and the team shifted to an IRB process that allowed participating hospitals to make CBEX available but without hospital research engagement. Hospitals could use their own IRB or the lead IRB to cover their participation and account for variability from the policies of the lead IRB. IRB changes were minimized to allow the project to proceed. An alternative IRB consent form was offered to hospitals that wanted to link the survey data to their own clinical data.	The team’s request to use existing database lists for patient recruitment was not feasible, making the team commit to in-person enrollment. The IRB was then initiated under the concept that staff at participating hospitals would not be engaged in research, and thus not be required to have IRB approval. The budget was not sufficient to pursue a more formal approach where clinic staff could be paid to actively seek out those who were eligible and flag charts for recruitment. Several hospitals were eventually able to implement recruitment efforts with antepartum or postpartum database lists approved by patient providers, and this approach was found to be more successful than in-person efforts.
Milestone 2: system design		
System design: survey Survey preparation required multiple iterations with respect to the following issues: Security issues/verification code Translate English to Spanish Desktop to mobile: Android/i-phones Use of e-mail vs phone (text message) Adapted CBEX to hospital requirements	Survey shortened, questions added, removed; effort to streamline interactions Team worked closely with IT vendor to make changes and refinements. Respondents without a phone and an email address could not participate due to security checks. Catholic hospitals removed survey questions regarding birth control. One hospital system (separate IRB) had specific new items they wanted included.	Monitoring of survey issues and dates of fixes was an important task. Survey design continued to evolve both because of use as patient-outcome measures and because of topical interest such as the pandemic or changes in childbirth services. A process will be required to track revisions and validation of new items. It remained unclear if email or text messages were more successful. Many patients did not use email (although email was required for sign-up) and text messages could be ignored or flagged as spam if patients were not expecting it.
System design: obtaining Data Patient registration and recruitment into the study was an ongoing concern.	Each hospital needed a different approach to recruitment to fit CBEX within the workflow. The lead hospital was able to recruit using postpartum discharge lists that were approved by patient physicians. One other hospital was also able to implement this strategy successfully. Range of recruitment strategies Flyers (with/without IRB numbers) Recruitment letter sent via patient portal Clinic Private physician office Hospital website Hospital Facebook page/Twitter page (social media) Pre-registration packet Childbirth ed classes GBS screening Antepartum fetal heart rate testing Triage Antepartum admission Prenatal appointment Postpartum appointment Birth navigators	Because of the different recruitment strategies required, there was a great deal of variability in the administration of the project at different hospitals.
System design: IT assistance IT vendor could not make changes to scope of work within existing budget as issues were uncovered at different hospital sites, including: Registration information and process Suitability of data collection platform for PHI linkage; PHI was kept separate from CBEX data. Extra charges incurred for out-of-scope changes	PI provided extra funding as necessary to achieve required changes in data collection process. 30 hours programming: $9,000 18 hours hacking repair: $5,400 5 hacking events: $1,065 in gift cards Total: $15,465	CBEX was placed on a third-party proprietary platform for data collection and all changes had to be made by the original vendor. The budget limited the team’s ability to move CBEX onto the vendor’s evolving platform. Study team has investigated and successfully programmed CBEX into 2 HIPPA compliant platforms and investigating a third option/vendor within the lead institution that has an automatic process for reach.
Milestone 3: system development and operations		
System development There were ongoing IT issues to be resolved, for example: Follow-up emails not programmed Potential to navigate away from survey before card redemption WiFi connection required in clinical recruitment areas	Program coordinator kept master list of all changes needed and bundled them by priority.	There was a need for an ongoing IT task list and an additional budget.
Operations Recruitment activities: staff involvement was difficult to develop and maintain. Staff were universally very busy. Fall/Winter holidays impeded recruitment.	Multiple strategies to improve recruitment were implemented and adapted to specific hospitals Used Target gift cards instead of Amazon Developed FAQ for hospital staff Had staff hand out referral cards Revised flyers to include QR codes and provided one-pager and video re how to use Provided gift tokens with CBEX logo for staff and patients to increase visibility and advertisement for the survey Lip balm with CBEX logo Button: Ask Me About CBEX Post-it with registration link Distributed document regarding how to register for CBEX Developed patient letters, invitations to participate Used Amazon tablets: required WiFi, ad removal, and programming with instructions to allow patients to take survey on tablet while awaiting appointment Used student volunteers to assist: they could get credit or letter of recommendation	IRB regulations did not allow staff to receive incentives to recruit, but marketing materials were permitted. Many staff did not see CBEX recruitment as part of their duties. Some staff were interested and continued to make patients aware of CBEX.
Milestone 4 ongoing implementation		
Activities Ongoing staff continuity problems, with staff on leave or changes in responsibilities Links to the survey were only given to patients, however, within this process, hacking of the program did occur, with people using false email addresses and phone numbers (together) to register as a different patient each time. The incentive was minimal ($5 per survey). “Postpartum only” recruitment was not able to be achieved by some hospitals. Covid brought a pause to all research activities for most of 2020, and an increased burden on all staff activity. There was a reduction of patients on tours/childbirth classes.	Lead hospital continued to provide assistance with potential recruitment techniques through site visits, personal assistance, and weekly group calls Hacking scenarios were detected and the affected links removed promptly by IT vendor. New links were issued. How the breaches occurred was not identified. The team began with antepartum recruitment activities and later folded in the “postpartum only” activities when it was determined that lead staff could focus on this distinct recruitment. Postpartum recruitment involved distinct activities and personnel from antepartum recruitment. After the pandemic started, the team shifted to online efforts for recruitment, including using the vendor’s implementation of online prenatal visits in participating hospitals to leverage recruitment activities. Antepartum testing remained a productive area for recruitment. The team provided hospitals with a slide set for accessing the survey as hospitals shifted to providing online prenatal content. An interim analysis was conducted to assist in maintaining stakeholder engagement. Partnership meetings allowed debriefing about COVID-19 events and impact within their hospitals and benchmark and provide emotional support to each other.	Recruitment continued to be the most concerning issue for all hospitals. The most successful recruitment strategies were through the antepartum testing area, where the patient spent time with staff who were often known to them, and through the database lists as described above. The use of volunteers and students was also successful until the onset of the pandemic. COVID-19 stimulated increased interest in online activities, and efforts were made to recruit patients in online prenatal visits, education, and childbirth classes. Many staff were especially burdened during the pandemic. Weekly and monthly partnership meetings continued even when research was halted. This remained a good avenue for stakeholders to voice concerns about the COVID-19 pandemic and its impact on childbirth services and patient experiences.
Targets Aggregate antepartum target and postpartum follow up target attained Hospital antepartum and postpartum responses were not adequate for analysis for 4 hospitals	Re-approached IRB for approval of using postpartum lists to recruit patients	The lead IRB allowed recruitment from electronic lists with health care provider approval. This approach was adopted by several hospitals and substantially increased postpartum recruitment.

*IT* information technology, *PHI* protected health information, *IRB* institutional review board, *BAA* business associate agreement, *CBEX* childbirth experience survey, *GBS* group B streptococcus test, *PI* principal investigator, *REDCap* research electronic data capture (REDCap), *FAQ* frequently asked questions, *QR* quick response code

### Milestone 1: Agreements

The original proposal for this project involved using electronic patient lists. It was intended that each site create a list of eligible patients for the third-party data collection company, which would initiate patient contact. Most hospitals were unable to pursue antepartum recruitment using such a list because they had paper-based pre-registration systems, with many pregnant patients remaining unregistered until they presented at the hospital for delivery. This compelled a switch to in-person antepartum recruitment. The lead IRB agreement was then established to allow “non-engagement in research” of the participating sites. Non-engagement means that participating hospital staff are limited to notifying patients regarding the study and does not allow for other research activities such as providing informed consent, administering incentives, or collecting protected health information (PHI) (U.S. Department of Health and Human Services, 2008).

### Milestone 2: System Design

The research team worked extensively with the sites to develop recruitment strategies that fit within site policies and workflow. Recruitment was dependent on staff motivation and individual interest. Student volunteers could be used at some hospitals to actively recruit, and they were instrumental to recruitment success; however, their participation ended after the start of the COVID-19 pandemic.

### Milestone 3: System Development/Operations

A variety of approaches were developed in collaboration with hospital staff to assist with recruitment. At some sites, staff were able to incorporate CBEX into their clinical duties, and such sites had higher recruitment rates. However, at other sites, staff involvement was difficult to develop and maintain.

### Milestone 4: Ongoing Implementation

Recruitment was interrupted due to the COVID-19 pandemic. When IRBs allowed research to resume, sites shifted to online recruitment due to ongoing concerns about social distancing and limiting the number of visitors in clinical spaces.

CBEX Part 2 was adapted for “postpartum only” recruitment. The lead hospital received approval to create lists of postpartum patients by provider name. Patients were contacted by e-mail with ~ 10% response rate. This approach was shared with other hospitals, some of which had success.

## Discussion

Hospital organizational capacity-building for the collection and sharing of PROs is a complex process, especially when attempted across independent hospitals with varying degrees of experience in research. The achievement of each implementation milestone requires a variety of different activities and leadership skills are essential to developing the workarounds that move organizations into this new territory.

Using the evidence presented in Table 2, the investigators identified a target for each of the capacities listed in the original framework (Korst et al., 2008) based on learning health system goals and suggested next steps for achieving them. This resulted in a capacity-based trajectory for the advancement of childbirth PRO data collection and sharing (Table 3). Descriptions of each of these targets and potential next steps to reaching the target are presented below.

**Table 3:** Trajectory matrix for hospital capacity building for sharing of patient-reported outcomes and experiences (PROs)

Capacities	Incentives	Leadership	Policies/IRB	Operating systems	IT	Legal aspects	Cross-collaboration	Patient impact & engagement
Current	External: call for Maternity HCAHPS, call for Birth Equity, widespread use of patient advisory groups	Developed several strong and motivated hospital teams	Achieved IRB approval but no organizational “engagement” and requirements varied by site	Designed recruitment strategies to fit organizations	Developed CBEX survey and third-party infrastructure to collect data	Legal involvement was limited to implementing BAA where necessary	Hospitals shared experiences with CBEX implement-tation and data collection strategies	Patients and patient advocates participated formally in collaborative
	Internal: Provided feedback regarding actionable items	Cooperated with several MFM/OB practices		Electronic systems used to recruit some patients	Potential for linkage to MRN approved for some		Hospitals shared perceived QI priorities	Team identified PROs and gaps in care linked to patient satisfaction
Obstacles	CBEX as another survey, no vision or priority for QI work	Lack of account-ability for data collection	CBEX viewed as research and not QI	Emphasis on in-person recruitment	Minimal experience with PRO data collection	Legal resources will be needed for data-sharing	Need to develop capacity for group problem-solving	Yet to implement interventions and track progress
Next steps	Develop vision and trajectory by getting to endgame for early adopters	Development of adminis-trative level champions	Transition CBEX from a research project to a QI project	Limit recruitment burden on staff	Integrate CBEX into virtual prenatal and postpartum visits	Begin discussions regarding goals, formal structure and leadership	Choose to improve a single PRO to demonstrate success	Develop “value” for patient feedback and participation within the organization
Next steps	Make more relevant by adapting to COVID-19; promote CBEX use with baseline data	Development of account-ability and high-level reporting	Formalize single IRB structure or integrate into organizational QI activities	Resources for local staff to develop and use electronic lists of eligible patients	Improve IT^j^ to allow self-identified early adopters to join and strengthen IT infrastructure for analysis and reports	Seek funding for collaborative infrastructure and develop rationale and avenues for sustainability	Develop hospital capacity for benchmarking and identifying effective interventions	Expand patient advisory groups within organizations and as part of collaborative
Target	Tracking PROs is integral to organization’s strategic plan	Patient experience reporting line (e.g., CXO)	PRO data collected as part of QI activities	Electronic processes in place to ID key populations	PRO integration or linkage to electronic health record	Formal collaborative governance structure	Successful and sustainable QI activities	Top-box patient satisfaction and formal role in collaborative

*HCAHPS* hospital consumer assessment of healthcare providers and systems, *IRB* institutional review board, *CBEX* childbirth experience survey, *BAA* business associate agreement, *MFM* maternal fetal medicine, *OB* Obstetric, *MRN* medical record number, *QI* quality improvement, *PRO*, patient-reported outcome, *IT* information technology, CXO: chief experience officer, *ID* identification, *Top-box* the most positive response to HCAHPS survey items (i.e., a score of “9” or “10”)

### Incentives

We posited that, under ***Incentives,*** the *target* is that the collection and use of PRO data be part of the hospital organization’s strategic goals (Cella et al., 2015). External incentives (e.g., federal revenue withholding tied to hospital satisfaction) (The Centers for Medicare & Medicaid Services, 2021) and internal incentives (e.g., improved patient satisfaction) can drive hospital organizations to develop meaningful collection and use of PROs (Korst et al., 2011). *A potential next step* to improve incentives to integrate PROs with the organization’s strategic goals would be to strengthen the vision of CBEX by demonstrating collaborative improvement in a single PRO and simultaneously demonstrating improved hospital satisfaction scores.

### Leadership

The *target *for the ***Leadership*** capacity is to reach the executive-level patient experience officer (CXO) within the organization. The CXO position demonstrates full accountability and support of the executive leadership to drive tangible improvement (Breen et al., 2021; Melder et al., 2020). *Potential next steps* to improve hospital leadership capacity would be (1) to implement CBEX activities under more formal organizational accountability such as subcontracts that provide a financial incentive or infrastructure with salary support or direct assistance with data collection; and (2) to promote CBEX results at higher organizational levels to encourage interest of those accountable for the patient experience.

### Policies/IRB

The *target* for the ***Policies/IRB*** capacity is to have CBEX data included in routine hospital QI activities. Suggested frameworks for learning health systems indicate that QI is a fundamental activity, and that research should be integrated into clinical practice (Damschroeder et al., 2009; Harrison & Grantham, 2018). Folding the collection of PRO data into routine QI activities would ease the burden of implementation (LeRouge et al., 2020), especially given that the risks from such data collection are minimal (Whicher et al., 2015). Revision of the IRB process to determine which QI activities require express prospective consent versus which may be addressed by routine disclosures is critical to the evolution and the future of PRO data collection and use (Finkelstein et al., 2015. *A potential next step* to improve hospital policies/IRB capacity would be to integrate CBEX into the hospital QI process.

### Operating Systems

The *target* for the ***Operating Systems*** capacity is to have electronic processes in place to identify and contact patients eligible for QI activities (Allen et al., 2021). If such processes are in place, then denominators for QI activities can be determined, patients can be contacted by either a third-party or the relevant hospital, and response rates tracked and improved. Although most participating hospitals did not have electronic lists of antepartum patients, postpartum electronic lists could be created from the EHR or hospital discharge data. *Potential next steps* to improve hospitals’ ability to participate in childbirth QI activities using electronic patient lists would be to provide resources to hospitals to create such lists and to define the primary activity of the CBEX project as QI to enable third-party patient contact.

### Information Technology

The *target* for the ***IT*** capacity is to have PRO data integrated with, or at least linked to, the EHR (Snyder et al., 2017). Given the variability in EHR sophistication in the hospitals likely to participate in a learning collaborative, a third-party data collection mechanism may be required, and indeed, advisable. For the CBEX project, several hospitals under one system received informed consent for collection of patient identifiers so that a link between CBEX and hospital data could be created. *Potential next steps* to improve PRO data collection and EHR linkage capacity would be (1) if relevant, structuring the informed consent to include patient notification of PHI collection and EHR linkage; (2) integrating CBEX recruitment into virtual antepartum and postpartum clinical appointment platforms; and (3) offering CBEX recruitment as a QI initiative that is compatible with clinical workflow.

### Legal

The *target* for the ***Legal Aspects*** capacity is to have a formal collaborative governance structure. Legal assistance is required to determine activities and resources to be used by the learning collaborative (Cella et al., 2015). The importance of legal services will be most relevant to later milestones required for such a collaborative, including expansion, governance, and sustainability. Given that legal views regarding data-sharing may vary widely by hospitals and hospital systems, *potential next steps* toward this target would be to achieve funding for collaborative activities and begin discussions regarding collaborative goals and formal relationships among the participants.

### Cross-Collaboration

The *target* for the ***Cross-Collaboration*** capacity is to demonstrate successful and sustainable collaborative activities. The development of the capacity of hospital staff to interpret data and determine optimal and responsive interventions is a critical component of learning health systems (Foley & Vale, 2017). *Potential next steps* toward the determination of priority QI activities and goal development, implementation of interventions, and data-tracking would be (1) to assure adequate baseline data collection for a priority topic; (2) to identify evidence-based interventions (if possible) or best practices that may address key priority concerns for active participants; and (3) to identify easily addressed environmental/service issues that have been reported by patients as problematic.

### Patient Impact and Engagement

The *target* for the ***Patient Impact and Engagement*** capacity is to demonstrate (1) improvement in PROs in the desired direction; and (2) ongoing participation in the collaborative process (Hartley & Seid, 2021; Key & Lewis, 2018; Rubin, 2017). It is important not only that patients provide feedback regarding services, but also that they should be active participants in the collaborative. If that participation is fulfilled, and hospitals respond, then, in theory, patient satisfaction should reach optimal levels. *Potential next steps* toward improving patient engagement capacity are to (1) develop increased organizational “value” for patient feedback and participation in QI activities (Fjeldstad et al., 2019); and (2) expand the use of patient advisory groups within hospitals and within the collaborative.

## Study Limitations

The context of this study was unique, and its generalizability to other healthcare conditions or environments may be limited. However, Table 3 may suggest potential avenues that could be employed by other collaboratives for achieving these suggested targeted capacities. Second, although 16 hospitals were approached based on existing relationships, the sample of hospitals represented diversity in patient populations limiting potential biases. Third, the COVID-19 pandemic undoubtedly impacted all organizational capacities, limiting resources and availability of staff to participate. However, it was the catalyst for virtual recruitment. Finally, an independent evaluator was not budgeted nor required as part of the funding award and this may limit the objectivity of the findings.

## Conclusion

This project demonstrates the complexity of hospital data-sharing for QI activities in the context of building a learning collaborative using PRO data. Different organizational capacities are important for each implementation milestone. This evaluation presents a trajectory that outlines the progress needed to bring the implementation to its full maturity and helps clarify next steps towards meeting those targets.

## Data Availability

Data sources used are listed in Table 1 (Data sources as evidence for the association between organizational capacities/incentives and project milestones), and Table 2 (Critical incidents, workarounds, and outcomes by milestones) provides the data that support the study findings.

## References

[CR2] Allen C, Coleman K, Mettert K, Lewis C, Westbrook E, Lozano P (2021). A roadmap to operationalize and evaluate impact in a learning health system. Learning health systems..

[CR3] Auffenberg GB, Qi J, Dunn RL, Linsell S, Kim T, Miller DC, Tosoian J, Sarle R, Johnston WK, Kleer E, Ghani KR, Montie J, Peabody J (2021). Evaluation of patient- and surgeon-specific variations in patient-reported urinary outcomes 3 months after radical prostatectomy from a statewide improvement collaborative. JAMA surgery.

[CR4] Bingham CO, Bartlett SJ, Merkel PA, Mielenz TJ, Pilkonis PA, Edmundson L, Moore E, Sabharwal RK (2016). Using patient-reported outcomes and PROMIS in research and clinical applications: experiences from the PCORI pilot projects. Quality of Life Research.

[CR5] Black N, Burke L, Forrest CB, Sieberer UH, Ahmed S, Valderas JM, Bartlett SJ, Alonso J (2016). Patient-reported outcomes: pathways to better health, better services, and better societies. Quality of Life Research.

[CR6] Breen W, Choi S, Hearld K, O'Connor SJ, Rafalski E, Borkowski N (2021). The association between an established Chief experience officer role and hospital patient experience scores. Patient Experience Journal.

[CR8] Cella, D., Hahn, E. A., Jensen, S. E., Butt, Z., Nowinski, C. J., Rothrock, N., & Lohr, K. N. (2015). *Patient-Reported Outcomes in Performance Measurement*. RTI Press. 28211667

[CR9] Damschroder LJ, Aron DC, Keith RE, Kirsh SR, Alexander JA, Lowery JC (2009). Fostering implementation of health services research findings into practice: a consolidated framework for advancing implementation science. Implementation Science.

[CR10] Finkelstein JA, Brickman AL, Capron A, Ford DE, Gombosev A, Greene SM, Iafrate RP, Kolaczkowski L, Pallin SC, Pletcher MJ, Staman KL, Vazquez MA, Sugarman J (2015). Oversight on the borderline: quality improvement and pragmatic research. Clinical Trials (london, England).

[CR12] Fjeldstad ØD, Johnson JK, Margolis PA, Seid M, Höglund P, Batalden PB (2019). Networked health care: Rethinking value creation in learning health care systems. Learning health systems.

[CR13] Foley TJ, Vale L (2017). What role for learning health systems in quality improvement within healthcare providers?. Learning health systems.

[CR14] Gensheimer SG, Wu AW, Snyder CF, PRO-EHR Users’ Guide Steering Group, & PRO-HER Users’ Guide Working Group (2018). Oh, the places we’ll go: patient-reported outcomes and electronic health records. The patient.

[CR15] Gregory KD, Korst LM, Saeb S, McCulloch J, Greene N, Fink A, Fridman M (2019). Childbirth-specific patient-reported outcomes as predictors of hospital satisfaction. American Journal of Obstetrics and Gynecology.

[CR16] Haider AH, Herrera-Escobar JP, Al Rafai SS, Harlow AF, Apoj M, Nehra D, Kasotakis G, Brasel K, Kaafarani HMA, Velmahos G, Salim A (2020). Factors Associated with long-term outcomes after injury: results of the functional outcomes and recovery after trauma emergencies (FORTE) multicenter cohort study. Annals of Surgery.

[CR17] Harrison MI, Grantham S (2018). Learning from implementation setbacks: Identifying and responding to contextual challenges. Learning health systems..

[CR18] Hartley DM, Seid M (2021). Collaborative learning health systems: Science and practice. Learning health systems.

[CR19] Helsten DL, Ben Abdallah A, Avidan MS, Wildes TS, Winter A, McKinnon S, Bollini M, Candelario P, Burnside BA, Sharma A (2016). Methodologic considerations for collecting patient-reported outcomes from unselected surgical patients. Anesthesiology.

[CR20] Key KD, Lewis EY (2018). Sustainable community engagement in a constantly changing health system. Learning health systems.

[CR21] Korst LM, Aydin CE, Signer JM, Fink A (2011). Hospital readiness for health information exchange: Development of metrics associated with successful collaboration for quality improvement. International Journal of Medical Informatics.

[CR22] Korst LM, Fridman M, Saeb S, Greene N, Fink A, Gregory KD (2018). The development of a conceptual framework and preliminary item bank for childbirth-specific patient-reported outcome measures. Health Services Research.

[CR23] Korst LM, Signer JM, Aydin CE, Fink A (2008). Identifying organizational capacities and incentives for clinical data-sharing: The case of a regional perinatal information system. Journal of the American Medical Informatics Association.

[CR24] Kowalski C, Roth R, Carl G, Feick G, Oesterle A, Hinkel A, Steiner T, Brock M, Kaftan B, Borowitz R, Zantl N, Heidenreich A, Neisius A, Darr C, Bolenz C, Beyer B, Pfitzenmaier J, Brehmer B, Fichtner J, Haben B, Dieng S (2020). A multicenter paper-based and web-based system for collecting patient-reported outcome measures in patients undergoing local treatment for prostate cancer: first experiences. Journal of patient-reported outcomes.

[CR25] Lavallee DC, Chenok KE, Love RM, Petersen C, Holve E, Segal CD, Franklin PD (2016). Incorporating patient-reported outcomes into health care to engage patients and enhance care. Health Affairs (project Hope).

[CR26] LeRouge, C., Austin, E., Lee, J., Segal, C., Sangameswaran, S., Hartzler, A., Lober, B., Heim, J., Lavallee, D.C. (2020). ePROs in Clinical Care: Guidelines and Tools for Health Systems. Accessed at https://epros.becertain.org/

[CR27] Marshall DA, Jin X, Pittman LB, Smith CJ (2021). The use of patient-reported outcome measures in hip and knee arthroplasty in Alberta. Journal of Patient-Reported Outcomes.

[CR28] Melder A, Robinson T, McLoughlin I, Iedema R, Teede H (2020). An overview of healthcare improvement: Unpacking the complexity for clinicians and managers in a learning health system. Internal Medicine Journal.

[CR29] Neale, J. (2008). Research Methods for Health and Social Care. Red Globe Press.

[CR30] Nordan L, Blanchfield L, Niazi S, Sattar J, Coakes CE, Uitti R, Vizzini M, Naessens JM, Spaulding A (2018). Implementing electronic patient-reported outcomes measurements: Challenges and success factors. BMJ Quality & Safety.

[CR31] Nilver H, Begley C, Berg M (2017). Measuring women’s childbirth experiences: A systematic review for identification and analysis of validated instruments. BMC Pregnancy and Childbirth.

[CR32] O'Connor MJ, Lorts A, Kwiatkowski D, Butts R, Barnes A, Jeewa A, Knoll C, Fenton M, McQueen M, Cousino MK, Shugh S, Rosenthal DN (2021). Learning networks in pediatric heart failure and transplantation. Pediatric transplantation.

[CR33] Rubin JC (2017). Patient empowerment and the Learning Health System. Learning health systems.

[CR34] Snyder C., Wu, A.W., (Eds). (2017). Users’ Guide to Integrating Patient-Reported Outcomes in Electronic Health Records. Baltimore, MD: Johns Hopkins University. 2017. Funded by Patient-Centered Outcomes Research Institute (PCORI); JHU Contract No. 10.01.14 TO2 08.01.15. http://www.pcori.org/document/users-guide-integrating-patient-reported-outcomeselectronic-health-record. Accessed 28 April 2022

[CR7] The Centers for Medicare & Medicaid Services (2021). The Hospital Value Based Purchasing (VBP) Program. Retrieved from https://www.cms.gov/Medicare/Quality-Initiatives-Patient-Assessment-Instruments/Value-Based-Programs/HVBP/Hospital-Value-Based-Purchasing

[CR35] U.S. Department of Health and Human Services (2008). Engagement of Institutions in Human Subjects Research. Retrieved from https://www.hhs.gov/ohrp/regulations-and-policy/guidance/guidance-on-engagement-of-institutions/index.html

[CR36] Wennberg, J. E., E. S. Fisher, and J. S. Skinner. 2002. “Geography and the Debate over Medicare Reform.” Health Affairs. Supplemental Web Exclusives: W96–114. 10.1377/hlthaff.w2.96 12703563

[CR37] Whicher D, Kass N, Saghai Y, Faden R, Tunis S, Pronovost P (2015). The views of quality improvement professionals and comparative effectiveness researchers on ethics, IRBs, and oversight. Journal of Empirical Research on Human Research Ethics.

[CR38] Yin, R. K. (2018). Case study research and applications: Design and methods. SAGE.

[CR39] Zheng H, Li W, Harrold L, Ayers DC, Franklin PD (2014). Web-based comparative patient-reported outcome feedback to support quality improvement and comparative effectiveness research in total joint replacement. EGEMS (Washington, DC).

